# Rapid Screening of Plastic-Degrading Enzymes Using an Optimized Cell-Free Protein Synthesis Platform

**DOI:** 10.4014/jmb.2503.03044

**Published:** 2025-07-14

**Authors:** SangKu Yi, Junhyeon Park, Jiyoung Park, Kyung-Jin Kim, Juhyun Kim

**Affiliations:** School of Life Science, BK21 FOUR KNU Creative BioResearch Group, Kyungpook National University, Daegu 41566, Republic of Korea

**Keywords:** Cutinase, PETase, cell-free protein synthesis

## Abstract

The accumulation of plastic waste poses a significant environmental challenge, necessitating the development of efficient plastic-degrading enzymes for bioremediation and recycling. However, traditional enzyme engineering approaches rely on microbial expression systems and are time-consuming and prone to unintended interactions between host cells and recombinant circuits. To address these limitations, a cell-free protein synthesis (CFPS) platform was developed for rapidly screening plastic-degrading enzymes. Using CFPS, cutinase and PET-degrading enzymes (PETases) were successfully synthesized, and their catalytic activities were assessed using polymer-containing agar plates. Clear degradation halos were observed for cutinase and PETase on polycaprolactone (PCL)-containing and bis (2-hydroxyethyl) terephthalate (BHET)-containing plates, respectively. The optimization of CFPS conditions revealed that enzyme synthesis efficacy was higher at room temperature than at 37°C. The screening of PETase variants (C3 N1377, Mipa-P, and C5 N1251), synthesized using the CFPS platform, demonstrated that the catalytic activity of Mipa-P was the highest and surpassed that of IsPETase. This finding was further validated through purified enzyme analysis. Our results establish CFPS as a rapid, scalable, and cell-free alternative platform for screening and optimizing plastic-degrading enzymes, facilitating advancements in enzymatic plastic recycling.

## Introduction

Plastics are widely used due to their durability, versatility, and cost-effectiveness; however, their accumulation in the environment has led to severe pollution and ecosystem disruption [1-3]. Conventional plastic waste management methods, including chemical recycling and incineration, are often energy-intensive processes that generate harmful byproducts [4-6]. Therefore, more sustainable, biological approaches are urgently required to facilitate plastic degradation.

Enzyme-driven biocatalytic depolymerization has emerged as a promising strategy for breaking down plastics into reusable monomers [7-10]. Among these enzymes, IsPETase, identified in *Ideonella sakaiensis*, efficiently degrades polyethylene terephthalate (PET) into its monomeric components—bis(2-hydroxyethyl) terephthalate (BHET), mono(2-hydroxyethyl) terephthalate (MHET), terephthalic acid (TPA), and ethylene glycol (EG)-which can be recycled into new plastic products [11, 12]. Also, a specific cutinase, TfCut2, exhibits hydrolytic activity on polycaprolactone (PCL), a biodegradable polyester [13, 14]. Despite their potential, the practical applicability of these enzymes is limited by their low catalytic efficiency, poor thermal stability, and high production costs in conventional microbial expression systems. To overcome these challenges, plastic-degrading enzymes have been engineered to improve catalytic performance, with bacteria systems frequently employed for gene cloning and enzyme expression [15-19]. However, this approach is labor-intensive and time-consuming, requiring multiple iterative cycles of genetic manipulation, enzyme production in living cells, and subsequent purification for screening [18, 20-22]. Moreover, heterologous gene expression in microbial hosts imposes a significant metabolic burden, often resulting in unintended mutations that further complicate the enzyme engineering process [23-25].

The Cell-Free Protein Synthesis (CFPS) system has emerged as a powerful alternative to traditional microbial approaches that can help overcome these limitations. Composed of crude cell extracts, energy sources, amino acids, and cofactors, CFPS provides a controlled and efficient platform for synthesizing target proteins, free from the constraints of living cells [26-28]. This setup enables the direct and efficient translation of genetic templates such as plasmid DNA, linear DNA, or mRNA, while avoiding common challenges associated with cellular systems such as resource competition and metabolic stress [29, 30]. Hence, CFPS offers several advantages over cell-based systems, including enhanced translation efficiency and rapid, flexible protein production. It also eliminates time-consuming processes such as cell growth, gene expression optimization, and protein purification, making CFPS an attractive tool for rapidly producing and screening engineered enzymes. By bypassing metabolic burdens and growth limitations, CFPS accelerates enzyme optimization and exhibits great promise for advancing plastic-degrading enzyme engineering [31].

In this study, a CFPS-based screening method was developed for screening plastic-degrading enzymes, including cutinase and PETases. Upon synthesizing these enzymes using the CFPS system, their catalytic activity was confirmed through clear halos formed on polymer-containing plates, indicating polymer degradation. The halo size was positively correlated with both the enzyme concentration and degradation efficiency. Conditions for efficient enzyme synthesis were optimized by evaluating template DNA concentrations and reaction temperature in the CFPS process. Our results highlight the potential of this simple and rapid CFPS-based screening platform for catalytic enzymes, overcoming the limitations associated with living cell systems. This approach facilitates the high-throughput screening and optimization of engineered plastic-degrading enzymes.

## Materials and Methods

### Bacterial Strains, Growth Conditions, and Plasmid Construction

All bacterial strains, plasmids, and primers used in this study are listed in Tables S1 and S2. *E. coli* strains were routinely grown at 37°C in Luria-Bertini (LB) broth (BD, USA) while shaking the solution at 180 rpm to achieve transformation and enzyme production. Kanamycin (50 μg/ml, Daejung, Republic of Korea) and ampicillin (150 μg/ml, Sigma-Aldrich, USA) were added to bacterial cultures when necessary.

### Construction of the Plastic Degrading Protein Expressions

The characteristics of the bacteria, plasmids, and primers used in this study are summarized in Tables S1 and S2. DNA manipulations were performed according to a standard protocol [32]. Plasmid DNA was isolated from bacterial cells using the Exprep Plasmid SV Mini Purification Kit (GeneAll, Republic of Korea, Cat. No. 101-102). Restriction endonucleases were purchased from New England Biolabs (NEB, USA). Plasmids expressing IsPETase, TfCut2, C3 N1377, Mipa-P, and C5 N1251 were previously built [33]. To construct a plasmid carrying the GFP-tagged cutinase, the coding sequences for GFP and cutinase (TfCut2) were amplified separately using Phusion High-Fidelity DNA Polymerase (NEB) with primer pairs FW/GFP RV for GFP and FW/TfCut2+Linker RV for cutinase. The resulting gene fragments were subsequently assembled with the pET28b vector using Gibson Assembly Master Mix (NEB) according to the manufacturer’s protocol.

For the preparation of DNA templates for CFPS, including TfCut2, TfCut2-GFP, C3 N1377, Mipa-P, and C5 N1251, PCR amplification was performed using Phusion High-Fidelity DNA Polymerase and primers T7 FW and T7 RV to incorporate the T7 promoter and T7 terminator into the PCR products.

The strains harboring plastic-degrading genes were pre-cultured overnight in LB medium and subsequently diluted 1:100 in fresh LB containing 20 μM isopropyl β-D-1-thiogalactopyranoside (IPTG; Thermo Scientific, USA) to induce protein expression under the T7-LacO promoter.

### Cell-Free Protein Synthesis System

The AccuRapid (Bioneer, Republic of Korea) Protein Expression kit was used as the Cell-Free protein synthesis (CFPS) system, with reagent quantities adhering to the manufacturer’s protocol. Concentrations of extracted plasmid and PCR product were measured using the NanoVue Plus spectrophotometer (GE Healthcare, USA), and an appropriate amount of DNA was added to CFPS solutions as the template DNA. CFPS mixtures were incubated for 2 h at 37°C or room temperature (RT). To minimize hindrance to enzyme activity, the CFPS reactant was dialyzed with distilled water using Slide-A-Lyzer MINI dialysis devices (10K MWCO, Thermo Fisher Scientific) for 10 min. The purified enzyme was spotted onto filter paper, from which 5-mm disks were prepared using a hole puncher. These disks were then incubated at room temperature for 2 h to evaluate catalytic activity.

### Preparation of Polymer Containing Media

Polymer-containing media were prepared following the protocol reported by Perez-Garcia *et al*. [34]. Briefly, 0.1 g of polycaprolactone (PCL, Average Mw~14000, Sigma-Aldrich, USA) was melted in 12 ml of acetone (Duksan, Republic of Korea) at 70°C. The PCL solution was then added to 200ml of LB or M9 minimal media (Sigma-Aldrich) containing 1.5% (w/v) agar. To prepare BHET-containing media, 0.15 g of BHET was dissolved in 6 ml of acetone at 70°C and added to 100 ml of M9 containing 1.5% (w/v) agar. To prepare PET-containing media, PET was ground to a particle size of < 200 μm. A total of 0.05 g of powdered PET was dispersed in 5 ml of 1,1,1,3,3,3-hexafluoro-2-propanol (Daejung). The solution was then slowly added dropwise into 50 ml of ice-cold water and stirred vigorously before mixing 100 ml of M9 medium containing 1.5% (w/v) agar media. BHET and PET powder were ground to a size < 200 μm before dissolution.

### Enzyme Purification

Enzyme purification was conducted as described by H. Seo *et al*. [33]. Briefly, an overnight-induced culture was harvested via centrifugation, and the cell pellet was resuspended in lysis buffer (40 mM Tris-HCl, pH 8.0) before being lysed by ultrasonication. After centrifugation (13,000 ×*g*, 30 min), the supernatant was loaded onto a Ni-NTA agarose column (Qiagen, Germany) pre-equilibrated with lysis buffer. Bound proteins were washed with 30 mM imidazole and eluted with 300 mM imidazole in lysis buffer. All purification steps were performed at 4°C.

### Measurement of Hydrolytic Activity

Hydrolytic activity was assessed based on the formation of clear halos after applying plastic-degrading enzymes onto polymer-containing agar plates. The size of each clear halo was quantified using an image analysis software ImageJ by considering the diameter of the clear zone surrounding each enzyme-spotted disc. The measured diameters were normalized to the diameter of the filter disc used for enzyme spotting. All experiments were performed in triplicate (*n* = 3), unless indicated otherwise, and data are presented as the mean ± standard deviation (SD). Statistical analyses were conducted using GraphPad Prism 10.4.2. Depending on the experimental comparison, statistical significance was evaluated using Student’s *t*-test or one-way ANOVA with Dunnett’s multiple comparison test. Differences were considered statistically significant at *P* < 0.05.

## Results and Discussion

### Instability of Heterologous Gene Expression in Living Systems

**Fig. 1:**
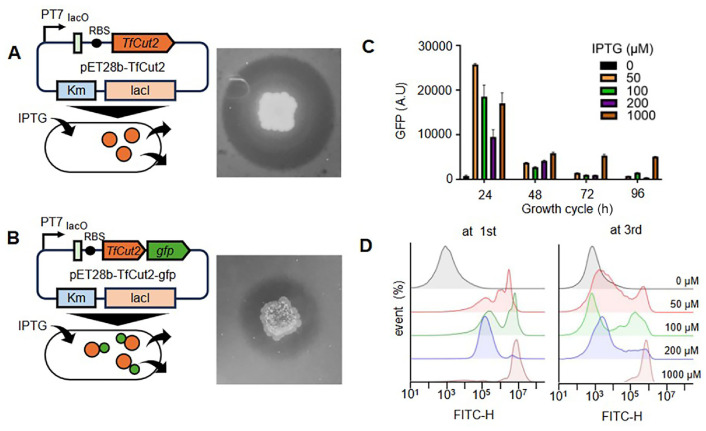
Expression of plastic degrading enzymes in a living system. (**A**) A plasmid carrying the IPTG-inducible *TfCut2* was constructed and introduced into *E. coli* BL21(DE3). When transformed cells were cultured on LB agar plates containing polycaprolactone (PCL) and 0.02 mM IPTG, the formation of a clear halo around cells indicated enzymatic hydrolysis activity. (**B**) PCL degradation was also observed in cells expressing the *TfCut2-gfp* fusion gene; however, hydrolysis activity was lower compared to the non-fused enzyme. (**C**) The stability of reporter activity across growth cycles was assessed by serially transferring the reporter strain by diluting cultures 100-fold into fresh medium containing varying IPTG concentrations, followed by incubation for 24 h. This process was repeated, and GFP activity was measured in each cycle using a microplate reader (*n* = 3) or (**D**) assessed at the 1^st^ and 3^rd^ cycles using flow cytometry analysis.

To assess the expression and catalytic activity of TfCut2 in bacteria, we constructed genetic circuits by cloning the gene encoding the plastic-degrading enzyme into the pET28b plasmid, which carried an IPTG-inducible T7 promoter system. The plasmid was introduced into *E. coli* BL21(DE3), a strain expressing T7 RNA polymerase. Since TfCut2 efficiently degrades polycaprolactone (PCL), transformed cells were cultured on PCL-containing plates with 100 μM IPTG to evaluate enzyme functionality. After incubating cells overnight, clear halos were formed around the cells, indicating successful enzyme synthesis, secretion, and subsequent PCL degradation (Fig. 1A).

While halo formation confirmed the functional expression and secretion of TfCut2, quantifying its expression level in living cells required an alternative approach. To address this issue, we fused TfCut2 to a green fluorescent protein (GFP) via a tandemly repeated linker (GGGGS 3) and assessed its catalytic activity and expression level. The fusion protein retained enzymatic function (Fig. 1B), although the halos produced by Tfcut2-GFP were less distinct than those formed by native cutinase. The use of GFP fusions to quantify TfCut2 expression introduced steric hindrance, reducing enzymatic activity [35]. Our preliminary results indicated that using a shorter linker substantially decreased hydrolytic activity. Thus, optimizing linker length, flexibility, or fusion orientation could effectively mitigate interference, preserving enzyme functionality while enabling accurate quantification.

To evaluate gene expression levels, fluorescence intensity was measured using a microplate reader. The reporter strain was cultivated in an LB medium with varying IPTG concentrations, and after 24 h, the highest GFP signal was observed at 50 μM IPTG (Fig. 1C). However, fluorescence intensity decreased at IPTG concentrations above 50 μM (Fig. 1C), suggesting that excessive induction may impose a metabolic burden on the cells. Nonetheless, these findings demonstrate that the genetic circuit can serve as a platform for evaluating plastic-degrading enzymes based on their expression levels in bacterial cells.

Since prolonged cultivation can affect heterologous gene expression, we monitored GFP expression across successive growth cycles. Serially transferring the reporter strain by diluting cultures 100-fold into fresh medium and incubating for another 24 h resulted in a significant decline in GFP intensity (Fig. 1C). This reduction persisted with further serial passage, with GFP activity remaining consistently low (Fig. 1C). To determine whether this decline was associated with genetic instability at the single-cell level, we analyzed the population via flow cytometry (Fig. 1D). Under the culture conditions used for microplate analysis, most cells exhibited strong GFP expression after 24 h (Fig. 1D). However, after two rounds of serial culture at 24-h intervals, a substantial portion of the population exhibited a loss in GFP expression, presumably due to the widespread mutations that had affected gene expression (Fig. 1D). Although population heterogeneity was not observed in samples treated with 1 mM IPTG, overall GFP expression levels were reduced, possibly due to the emergence of mutations in the reporter's expression or regulatory system (Fig. 1D). These findings highlight that continuous induction of heterologous gene expression in living systems often leads to instability, driven by cellular burden and genetic mutations. The progressive decline in expression levels and increased cell-to-cell variability during extended cultivation poses significant challenges for the reliable screening and characterization of plastic-degrading enzymes. This variability necessitates a more stable expression system. To address these limitations, we explored cell-free protein synthesis (CFPS) as an alternative. Unlike living systems, CFPS eliminates cellular stress and unintended mutations associated with gene expression burdens [29, 30]. Leveraging these advantages, we established CFPS as a robust platform for screening plastic-degrading enzymes.

### Validation of Catalytic Enzyme Productivity of CFPS Using Cutinase

**Fig. 2:**
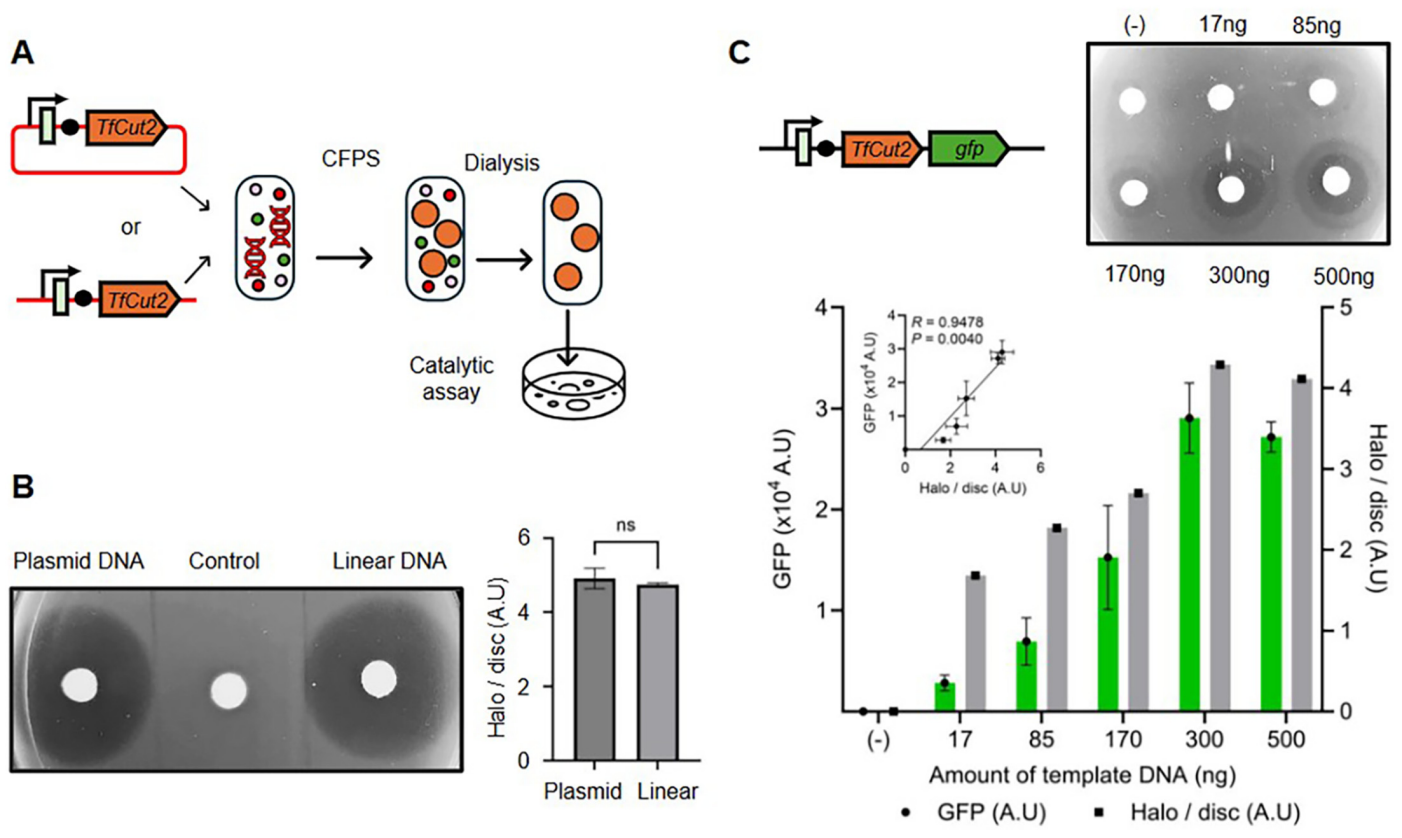
CFPS-based TfCut2 synthesis and its hydrolysis activity. (**A**) Schematic representation of the CFPS workflow. Reactions were allowed to occur using equal concentrations (0.15 pmol) of a DNA template encoding *TfCut2* under the T7 promoter, provided as either plasmid or PCR-amplified linear DNA sequences. Following a 2-h incubation at 37°C, reaction mixtures were dialyzed for 10 min to remove small molecules. (**B**) CFPS products were applied to M9 agar plates with PCL using filter paper to limit diffusion. After incubating for 2 h at 37°C, clear degradation halos were observed, indicating enzymatic activity (*n* = 3). Comparison of relative halo size showed no significant difference between reactions using plasmid DNA and linear DNA templates (ns, not significant; *p* > 0.05). (**C**) Using the same approach, GFP-fused TfCut2 was synthesized with varying amounts of linear DNA, and both GFP fluorescence intensity and catalytic activity were measured (*n* = 3). A positive correlation was observed between GFP expression level and halo size, as determined by Pearson correlation analysis (r = 0.9478, *P* = 0.0040). (**B, D**) Error bars represent S.D. CFPS, Cell-free protein synthesis.

To evaluate the effectiveness of cell-free protein synthesis (CFPS) for screening catalytic enzymes, we synthesized TfCut2 using a commercially available CFPS kit and assessed its catalytic activity through a polycaprolactone (PCL) degradation assay previously optimized for TfCut2-expressing cells. CFPS reactions were conducted using identical concentrations of DNA templates encoding TfCut2 under the control of the T7 promoter, consisting of plasmid DNA or PCR-amplified linear DNA (Fig. 2A). To ensure an accurate and fair comparison between the different DNA templates, we introduced an equal molar quantity (0.15 pmol) of each DNA type into the CFPS reaction. Using equal molar concentrations guarantees that each reaction contains the same number of template molecules, thereby avoiding discrepancies that could result from variations in DNA length. Following a 2 h incubation period at 37°C to synthesize the protein, reaction mixtures were dialyzed against distilled water for 10 min to remove small molecules potentially interfering with enzymatic assays. Aliquots of 50 μl were obtained from each dialyzed sample and applied onto M9 agar plates supplemented with PCL, using filter papers to confine samples and prevent diffusion. After incubation at 37°C for 2 h, clear degradation halos were observed on PCL-containing plates, indicating enzymatic activity. Importantly, halo sizes and clarity were comparable between plasmid-derived and PCR-amplified DNA templates (Fig. 2B), suggesting no disadvantage in using PCR-amplified DNA. Due to these equivalent results, subsequent CFPS assays exclusively utilized PCR-amplified DNA templates, offering advantages such as cost-effectiveness and convenience.

Next, we investigated how varying the DNA template amount influenced protein yield and catalytic activity in CFPS reactions. Using a TfCut2-GFP fusion construct allowed the parallel measurement of fluorescent and catalytic activities across multiple DNA template concentrations. CFPS reactions were conducted as described above, products were transferred into 384 well plates, and GFP fluorescence was quantified using a microplate reader. Fluorescence intensity increased proportionally with DNA template quantities of up to 300 ng but plateaued at higher concentrations (500 ng; Fig. 2C). Similarly, when CFPS-produced cutinase was evaluated via application onto PCL agar plates, the observed halo size and clarity correlated closely with DNA template amounts, aligning effectively with GFP fluorescence measurements (Fig. 2C). The observed increases in fluorescence intensity and halo formation with higher amounts of DNA template - up to 300 ng - indicate that protein production and enzymatic activity in CFPS are positively correlated with template DNA concentration. These findings collectively demonstrate that CFPS is an effective platform for rapidly synthesizing and screening catalytically active enzymes, with enzymatic functionality readily verified through substrate-containing agar assays. Crucially, optimizing DNA template concentrations is essential to maximize protein yield and catalytic efficiency during CFPS reactions, with optimal conditions likely varying across different enzymes, depending on the specifically targeted enzyme.

### Efficiency Screening of the PETase Variants Using CFPS

To expand the applicability of the CFPS-based enzyme screening platform initially established with Cutinase, we evaluated its effectiveness for producing and assessing PETases. While both cutinase and PETase function as catalytic enzymes, their distinct substrate preferences affect their potential for degrading plastic. Cutinase primarily hydrolyzes low-crystallinity polyesters such as PCL, a relatively simple substrate that facilitates straightforward activity detection. In contrast, PETase targets polyethylene terephthalate (PET), a highly crystalline polyester with aromatic ring structures that confer considerably more resistance to enzymatic degradation [36]. Given the escalating global plastic pollution crisis and the need for sustainable degradation solutions, PETase has garnered significant interest for its ability to degrade PET under mild conditions [37]. To enhance PETase catalytic efficiency, several engineered PETase variants have been developed [16, 38]. However, screening and optimizing PETase variants for improved activity, stability, and substrate specificity remain major bottlenecks, limiting their practical applicability in industrial and environmental settings. A CFPS-based approach, if successfully applied to PETase, could provide a high-throughput, cell-free alternative to traditional expression systems, enabling the rapid discovery and optimization of PET-hydrolyzing enzymes without the constraints associated with cell viability, secretion pathways, or complex expression conditions.

**Fig. 3:**
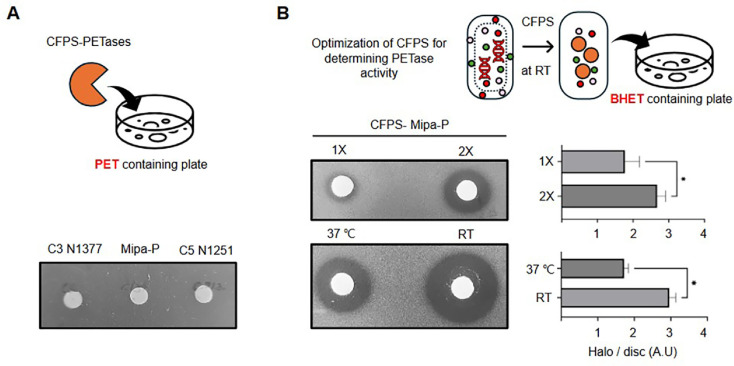
Optimization of CFPS reactions for PETase synthesis. (**A**) PETase variants (C3 N1377, Mipa-P, and C5 N1251) were synthesized using CFPS, and their hydrolysis activity was assessed on PET-containing plates (*n* = 3). No detectable halo formation was observed. (**B**) To further evaluate the hydrolysis activity of a specific PETase, PET was replaced with its soluble analog, BHET, as the substrate. Additionally, the CFPS reaction volume was increased (2X), and the CFPS synthesis was performed at room temperature (RT) and 37°C to optimize PETase production (*n* = 3). Mipa-P produced under optimized CFPS conditions (2X reaction volume and RT synthesis) generated significantly larger halos, indicating enhanced catalytic activity. Asterisks indicate significant differences established by an unpaired two-tailed *t*-test (*P* < 0.05). BHET, bis(2- hydroxyethyl) terephthalate.

Considering these advantages, we aimed to test the feasibility of CFPS for synthesizing and screening PETases and evaluated its broader applicability for enzymes targeting structurally complex and industrially relevant substrates. Thus, we conducted a comparative analysis of PETase variants within the CFPS framework, to evaluate their activity and stability. We synthesized three PETases (C3 N1377, Mipa-P, C5 N1251) [33] using CFPS and assessed their catalytic activity on PET-containing agar plates, following the approach used for cutinase. However, none of the CFPS-expressed PETases exhibited detectable catalytic activity against PET (Fig. 3A). This lack of activity was probably attributable to the buffer conditions used during CFPS synthesis and dialysis, which may have hindered the interaction of PETase with PET. In addition, the rigid and crystalline structure of PET presents a physical barrier to enzymatic access, typically requiring a high local concentration of active enzyme for effective hydrolysis. Given the moderate expression levels typically obtained through CFPS, the resulting concentration of PETase may not have reached the threshold necessary to effectively hydrolyze the highly crystalline PET surface, thereby preventing visible degradation. Therefore, we used bis(2-hydroxyethyl) terephthalate (BHET), a soluble monomeric substrate, to assess catalytic activity under more permissive conditions. Mipa-P [33] was synthesized via CFPS, and its activity was tested on BHET-containing plates. When prepared using the same approach as cutinase, clear halo zones appeared around enzyme-containing disks (Fig. 3B), confirming their enzymatic activity. Furthermore, doubling CFPS reaction mixture volumes resulted in larger halos, indicating increased PETase production in CFPS (Fig. 3B). To further enhance CFPS-based PETase synthesis, we optimized incubation conditions by reducing reaction temperature and increasing reaction volume. Since CFPS relies on T7 RNA polymerase derived from *E. coli*, which functions optimally at 37°C, reactions are typically conducted at this temperature. Although the overall capacity for protein synthesis may be reduced at room temperature, the PETase synthesized under these conditions exhibited significantly enhanced hydrolytic activity (Fig. 3B), likely attributable to improved protein folding, stability, and functional integrity [39, 40]. Collectively, these adjustments resulted in notably larger halos on BHET-containing plates. These findings underscore the potential for optimizing CFPS reaction parameters to enhance enzyme production and activity. With appropriate modifications, CFPS provides a powerful platform for screening and engineering PET-degrading enzymes, accelerating the development of highly efficient biocatalysts for plastic degradation.

### Application of CFPS-Based Screening to Evaluate Engineered PETase Variants

**Fig. 4:**
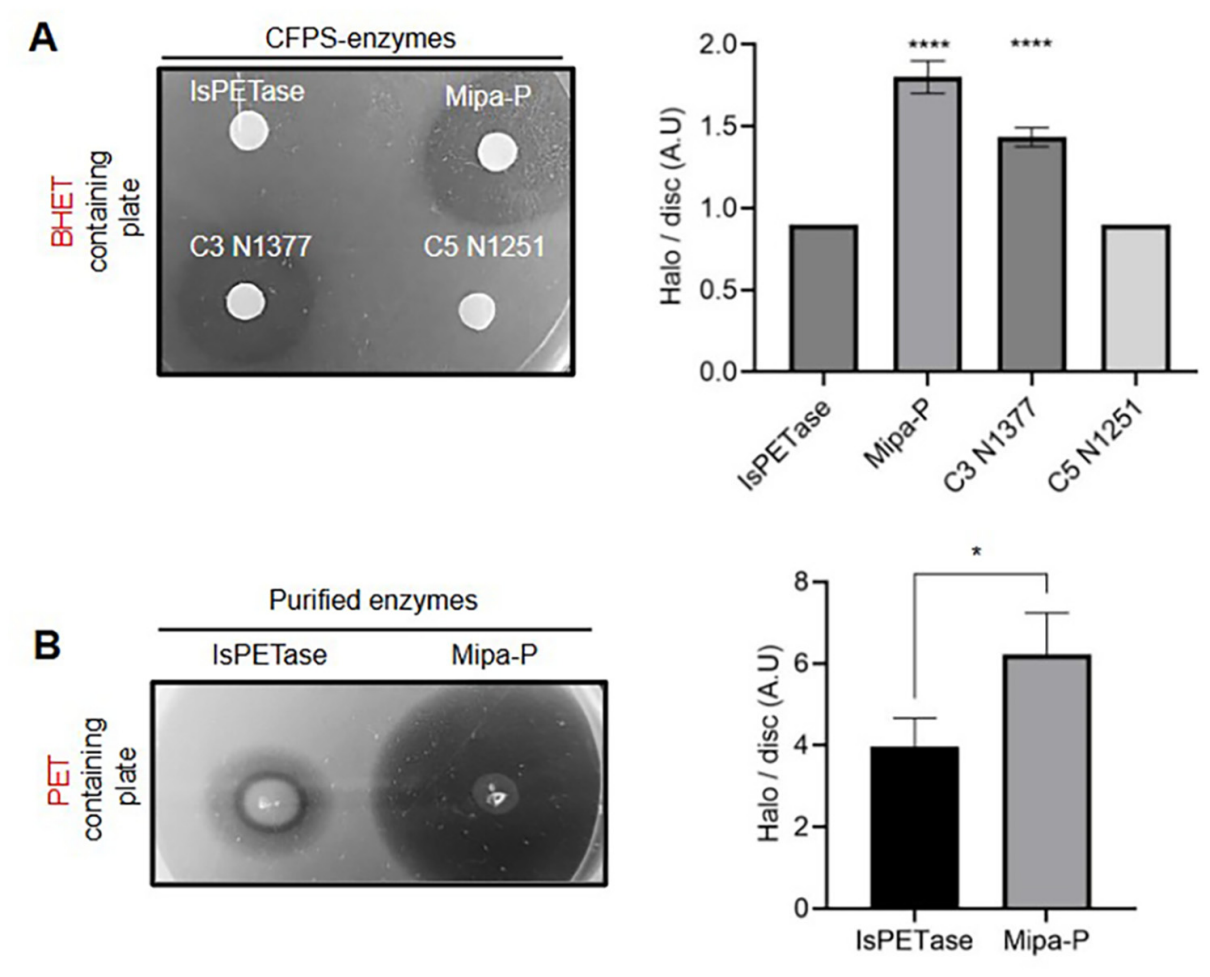
Screening hydrolysis activity of PETase variants synthesized via CFPS. (**A**) PETase variants (IsPETase, C3 N1377, Mipa-P, and C5 N1251), synthesized via CFPS, were assessed for enzymatic activity using BHET as a substrate. Halo formation served as an indicator of relative catalytic activity. A quantitative comparison using IsPETase as a reference enzyme revealed that engineered Mipa-P exhibited the highest enzymatic activity, followed by C3 N1377, while IsPETase and C5 N1251 demonstrated minimal activity as same as reference (*n* = 3). Statistical significance was determined using one-way ANOVA followed by Dunnett’s multiple comparisons test against the IsPETase. Both Mipa-P and C3 N1377 showed significantly higher activity compared to IsPETase (**** *P* < 0.0001). (**B**) To prove these findings, extracted and purified Mipa-P and IsPETase from *E. coli* were applied to PET-containing plates to compare catalytic efficiency (*n* = 3). The results confirmed that Mipa-P exhibited superior activity to IsPETase, consistent with CFPS-based screening results. This was further supported by quantitative analysis of halo size, which showed a significant increase in degradation area for Mipa-P (unpaired two-tailed *t*-test, *P* < 0.05).

Building upon the optimization of CFPS-based enzyme synthesis and substrate selection for PETase, we sought to demonstrate its practical application in evaluating engineered PETase variants. To achieve this, we synthesized three efficiency-verified PETase variants using the optimized CFPS-based method and evaluated their catalytic activity on BHET-containing plates for comparative screening. The resulting halo sizes varied based on the catalytic efficiency of each PETase (Fig. 4A). Mipa-P exhibited the highest activity, forming the largest halos, followed by C3 N1377, whereas both IsPETase and C5 N1251 showed no detectable activity. Additionally, we compared the halo-forming PETases (Mipa-P and C3 N1377) with IsPETase as a reference to evaluate their relative catalytic performance. This assessment facilitated a relative comparison of the efficiency of engineered PETase variants against IsPETase, the naturally occurring enzyme used for plastic degradation (Fig. 4B). The results demonstrated that Mipa-P exhibited robust enzymatic activity, generating clearer and larger halo zones on BHET-containing media than any of the other hydrolases tested. Notably, we observed minimal hydrolytic activity for IsPETase and C5 N1251 using this CFPS platform, despite their reported plastic-degrading capabilities [33]. This finding suggests that only enzymes possessing a sufficiently high level of intrinsic hydrolytic activity can be effectively screened with the current CFPS method. Overcoming this limitation will likely require further optimization of both the CFPS conditions and the screening methodologies.

To validate CFPS-based screening results, the catalytic activity of purified Mipa-P and IsPETase was further confirmed using PET-containing media (Fig. 4B). Switching from BHET to PET substrates with purified enzymes allowed us to directly validate enzyme activity on a more relevant polymer, emphasizing the platform’s capability for rapid screening and deployment of engineered enzymes. Furthermore, this experiment enabled a direct comparison of enzymatic efficiency under controlled conditions, reinforcing the trends observed in the CFPS-based screening (Fig. 4A). These results highlight the potential of CFPS as an efficient platform for screening and evaluating engineered PETase variants. By bypassing traditional bottlenecks associated with protein expression, CFPS provides a valuable tool for rapidly identifying and optimizing plastic-degrading enzymes, paving the way for future advancements in enzymatic plastic degradation strategies.

## Conclusion

The instability of heterologous gene expression in living systems poses a significant challenge to enzyme characterization and stable production. The prolonged cultivation of *E. coli* expressing TfCut2 led to a sharp decline in expression levels, likely due to cellular burden and accumulated genetic mutations. This study demonstrates that CFPS offers a stable and efficient platform for screening plastic-degrading enzymes, overcoming the limitations of conventional microbial expression systems. We successfully synthesized and evaluated TfCut2 and PETase variants, confirming their catalytic activity on PCL- and BHET-containing plates, respectively. Notably, the optimization of CFPS reaction conditions, including the reduction in incubation temperature and increase in reaction volume, enhanced enzyme stability and production. Although direct PET degradation was not observed, CFPS-based screening with BHET effectively helped differentiate PETase variants by their hydrolysis activity. These findings establish CFPS as a powerful tool for enzyme engineering, enabling the rapid screening and optimization of plastic-degrading biocatalysts for bioremediation and industrial recycling.
